# Human olfaction: odour coding and cross-modal concept representation in single olfactory cortex neurons

**DOI:** 10.1038/s41392-024-02073-y

**Published:** 2024-11-27

**Authors:** Antonia Beiersdorfer, Markus Rothermel, Christian Lohr

**Affiliations:** 1 Institute of Cell and Systems Biology of Animals, University of Hamburg, Hamburg, Germany; 2 Institute of Physiology, RG Neurophysiology and Optogenetics, Medical Faculty, Otto-von-Guericke-University, Magdeburg, Germany

**Keywords:** Olfactory system, Cellular neuroscience

In a recent study published in *Nature*, Kehl et al.^1^ demonstrate that single neurons in humans specifically respond to the smell of different odours and encode stimulus properties, such as odour identity and valence. Surprisingly, a subpopulation of odour-modulated neurons in the piriform cortex (PC) and amygdala also responds to odour-related images and texts, suggesting cross-modal conceptual representations.

**Fig. 1:**
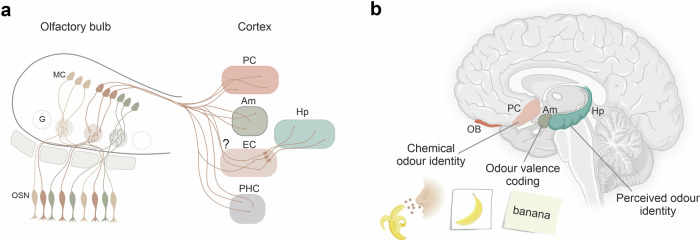
Anatomical and functional properties of the olfactory cortex. **a** Mitral cells (MC) in the olfactory bulb receive input from olfactory sensory neurons (OSN) in the glomeruli (G) and project to the piriform cortex (PC), amygdala (Am), entorhinal cortex (EC) and parahippocampal cortex (PHC). While direct synaptic input from the olfactory bulb into the EC is well established in rodents, it has not been confirmed in humans (?). The hippocampus (Hp) receives odour information from the EC. **b** Kehl et al.^1^ demonstrated that neurons in the PC decode chemical odour identity, while neurons in the amygdala decode odour valence and neurons in the hippocampus decode perceived odour identity. A subpopulation of neurons in the PC and Am responds to a particular odour (for example the smell of banana) as well as an image related to the odour (a picture of a banana) and odour-related text (the written word “banana”). OB, olfactory bulb. Created with Biorender.com and CorelDraw

The current understanding of olfactory perception is primarily derived from animal studies.^2^ Olfaction starts with detecting odourants in the nasal cavity through olfactory sensory neurons (OSNs), each expressing one out of ~350 functional olfactory receptors in humans (~1300 in mice) and projecting its axon to the olfactory bulb. Axons of OSNs that express the same receptor converge in specific regions known as glomeruli (Fig. 1a). A topographic representation of activated glomeruli—the olfactory map—encodes odour identity. However, this topographic representation dissolves along the olfactory axis, with olfactory bulb output neurons diverging to connect with widely dispersed neurons within the PC and medial temporal lobe (MTL) regions such as the entorhinal cortex (EC), parahippocampal cortex (PHC), hippocampus and amygdala.^3^ This scattered cortical representation of olfactory information complicates the investigation of neuronal circuits underlying odour perception in humans using non-invasive electrophysiology (e.g. EEG) and imaging techniques (e.g. fMRI), which have a low temporal and spatial resolution. This has long resulted in a knowledge gap between animal and human olfaction research.

Kehl et al.^1^ recorded multiple individual neurons in the PC and MTL of epilepsy patients undergoing pre-surgical invasive seizure monitoring with implanted electrodes. Data were collected from a limited set of patients, and this cohort might not represent humans in general, in particular considering the role of the PC in seizure physiology.^4^ The authors recorded over 2000 neurons and identified single units whose firing rate increased in response to odour presentation in the PC, amygdala, EC and hippocampus, while no change in firing rate was observed in the PHC. Compared with the olfactometers typically used in rodent research, the odour pens used here have a lower temporal resolution, and faster dynamics might not have been captured. Notably, odour representation was broader in the PC than in the MTL, with 40% of PC neurons being odour-modulated, compared to only 20% or fewer in MTL regions. On the other hand, the amygdala and hippocampus showed the highest degree of population sparseness. The number of neurons sampled is inevitably low, and there are limitations when trying to draw even relative conclusions about sparse coding between regions. A machine learning algorithm was trained to identify odours from the neural data (classifier analysis) and predict odour identity from the neuronal activity. The highest decoding performance was observed in the PC, underscoring its putative role in odour identity coding (Fig. 1b). A limitation might be that even if decoding performance correlates with odour identity and valence, that does not mean that the brain regions examined do in fact encode odour identity or valence. Repetitive odour presentations significantly decreased the response strength of individual neurons recorded in the PC, amygdala and hippocampus. This suppression most likely reflects central habituation processes, as the 5-minute interval of repeated odour presentations far exceeds the peripheral adaptation duration observed in OSNs.^2^

The amygdala, EC and hippocampus are associated with emotional responses and memory formation, providing a neural basis for emotional reactions and memory retrieval. The authors asked how far these brain areas are involved in odour identification and valence ratings. Participants rated odour pleasantness during the presentation, and the obtained valence ratings were used to sort neuronal responses. Odour-modulated neurons in the amygdala exhibited significantly stronger responses to liked versus disliked odours, a correlation not observed in other brain regions. Since odours of positive valence were overrepresented in the panel, however, it is not clear how generalisable the results are to negative or neutral valence odours. Participants were also asked to identify the presented odours, requiring odour perception, recognition, and recall of the semantic odour label. Accurate odour identification was correlated with increased firing rates of odour-modulated neurons. Interestingly, a correlation between odour identification performance and odour decoding accuracy was, however, only observed in the hippocampus. The authors also examined how neurons decode chemical (i.e. real) vs. the perceived (i.e. subjective perception based on selected odour labels) odour identity. While PC neurons reliably decoded chemical identity, hippocampal neuron activity predicted subjectively perceived identities; when incorrect labels were assigned, hippocampal neurons decoded the perceived (incorrect) rather than the chemical (correct) identity.

Our senses do not operate in isolation, and information from different sensory modalities is combined to form a coherent internal representation of the environment. The authors analysed possible cross-modal integration of visual and olfactory cues and identified neurons responsive to the presentation of images related to specific odours. Image decoding accuracy was highest in the PC, demonstrating that PC neurons are not exclusively driven by odours, arguing for associative functions in PC, as shown in animal models.^5^ When classifiers trained on images attempted to predict odour identity, they succeeded for both the PC and amygdala. In contrast, those trained on odours could only predict corresponding images for the amygdala, demonstrating that amygdala neurons might generalise their coding between the olfactory and visual domains. Finally, the authors provide the first evidence for semantic coding of olfactory information by demonstrating olfactory 'concept' cells. Neurons in the PC and the amygdala showed responses not only to a specific odour but also to an image depicting that odour and to the written word for that odour (i.e. the smell of a banana, an image of a banana and the name 'banana') (Fig. 1b). In how far subjects could have imagined smells when prompted by visual cues is not clear. Potentially, imagined odours could activate odour-sensitive neurons in, e.g. piriform cortex without the need for cross-modal integration.

In summary, Kehl et al.‘s pioneering study^1^ offers new insights into human odour perception at the single-neuron level, narrowing the gap between animal and human olfactory research. Different functions are associated with different olfactory cortical areas: PC neurons excel at decoding chemical odour identity; amygdala neurons predict odour valence; and hippocampal neurons are linked to subjectively perceived odour identity. Additionally, neurons responsive to odours, related images, and texts suggest the presence of cross-modal conceptual representations of odours within olfactory cortical regions. While this study reveals some parallels to animal research, it does not address the inherent limitations of human studies, such as the challenges of pharmacological and genetic modifications. Future investigations should assess whether the methodologies employed by Kehl et al. and their findings can be leveraged to enhance the diagnosis and treatment of temporal lobe epilepsy, particularly in the identification of seizure foci prior to surgical intervention.
